# Limitations of microscopy to differentiate *Plasmodium* species in a region co-endemic for *Plasmodium falciparum*, *Plasmodium vivax* and *Plasmodium knowlesi*

**DOI:** 10.1186/1475-2875-12-8

**Published:** 2013-01-08

**Authors:** Bridget E Barber, Timothy William, Matthew J Grigg, Tsin W Yeo, Nicholas M Anstey

**Affiliations:** 1 Menzies School of Health Research and Charles Darwin University, Darwin, Northern Territory, Australia; 2 Infectious Diseases Unit, Queen Elizabeth Hospital, Kota Kinabalu, Sabah, Malaysia; 3 Sabah Department of Health, Kota Kinabalu, Sabah, Malaysia; 4 Division of Medicine, Royal Darwin Hospital, Darwin, Northern Territory, Australia

**Keywords:** *Plasmodium knowlesi*, Malaria, Microscopy, Diagnosis

## Abstract

**Background:**

In areas co-endemic for multiple *Plasmodium* species, correct diagnosis is crucial for appropriate treatment and surveillance. Species misidentification by microscopy has been reported in areas co-endemic for vivax and falciparum malaria, and may be more frequent in regions where *Plasmodium knowlesi* also commonly occurs.

**Methods:**

This prospective study in Sabah, Malaysia, evaluated the accuracy of routine district and referral hospital-based microscopy, and microscopy performed by an experienced research microscopist, for the diagnosis of PCR-confirmed *Plasmodium falciparum*, *P*. *knowlesi*, and *Plasmodium vivax* malaria.

**Results:**

A total of 304 patients with PCR-confirmed *Plasmodium* infection were enrolled, including 130 with *P*. *knowlesi*, 122 with *P*. *falciparum*, 43 with *P*. *vivax*, one with *Plasmodium malariae* and eight with mixed species infections. Among patients with *P*. *knowlesi* mono-infection, routine and cross-check microscopy both identified 94 (72%) patients as “*P*. *malariae*/*P*. *knowlesi*”; 17 (13%) and 28 (22%) respectively were identified as *P*. *falciparum*, and 13 (10%) and two (1.5%) as *P*. *vivax*. Among patients with PCR-confirmed *P*. *falciparum*, routine and cross-check microscopy identified 110/122 (90%) and 112/118 (95%) patients respectively as *P*. *falciparum*, and 8/122 (6.6%) and 5/118 (4.2%) as “*P*. *malariae*/*P*. *knowlesi*”. Among those with *P*. *vivax*, 23/43 (53%) and 34/40 (85%) were correctly diagnosed by routine and cross-check microscopy respectively, while 13/43 (30%) and 3/40 (7.5%) patients were diagnosed as “*P*. *malariae*/*P*. *knowlesi*”. Four of 13 patients with PCR-confirmed *P*. *vivax* and misdiagnosed by routine microscopy as “*P*. *malariae*/*P*. *knowlesi*” were subsequently re-admitted with *P*. *vivax* malaria.

**Conclusions:**

Microscopy does not reliably distinguish between *P*. *falciparum*, *P*. *vivax* and *P*. *knowlesi* in a region where all three species frequently occur. Misdiagnosis of *P*. *knowlesi* as both *P*. *vivax* and *P*. *falciparum*, and *vice versa*, is common, potentially leading to inappropriate treatment, including chloroquine therapy for *P*. *falciparum* and a lack of anti-relapse therapy for *P*. *vivax*. The limitations of microscopy in *P*. *knowlesi*-endemic areas supports the use of unified blood-stage treatment strategies for all *Plasmodium* species, the development of accurate rapid diagnostic tests suitable for all species, and the use of PCR-confirmation for accurate surveillance.

## Background

Despite recent progress towards elimination, malaria continues to affect over 200 million people per year with an estimated 655,000 deaths [1]. Although most deaths are caused by *Plasmodium falciparum*, the relative contribution of the non-falciparum *Plasmodium* species to the global malaria burden is increasing as incidence of *P*. *falciparum* falls [2-4]. The most widely distributed of these species is *Plasmodium vivax*, which accounts for half of the world’s malaria and is increasingly recognized as a cause of severe and potentially fatal disease [5-8]. Moreover, reducing transmission of *P*. *vivax* has proved more difficult than *P*. *falciparum*, with successful malaria control programmes in some countries leading to an increase in incidence of *P*. *vivax* as overall malaria rates drop [2,3]. More recently, the simian parasite *Plasmodium knowlesi* has been identified as the most common cause of human malaria in parts of Malaysia [9-14], with its emergence also associated with reduction in incidence of the human *Plasmodium* species [12]. *Plasmodium knowlesi* is capable of causing severe disease and death [9,13,15-18], and is increasingly reported in other Southeast Asian countries [19].

In areas co-endemic for *P*. *falciparum*, *P*. *vivax* and *P*. *knowlesi*, species-differentiation at the time of diagnosis is crucial for directing appropriate treatment, particularly in settings which have separate treatment policies for different species, most commonly artemisinin-combination treatment (ACT) for *P*. *falciparum* and chloroquine for non-falciparum species. Even in regions such as Papua, Indonesia, which have adopted a unified treatment strategy of ACT for all malaria [1], diagnosis of *P*. *vivax* is still required to allow administration of anti-hypnozoite treatment to prevent relapses, with misdiagnosis of this species potentially leading to increased morbidity and transmission. In areas also endemic for *P*. *knowlesi*, accurate diagnosis is important for epidemiological surveillance of this potentially fatal emerging zoonotic infection.

Microscopy of stained blood smears remains the standard method of malaria diagnosis in most parts of the malaria-endemic world, and ideally allows differentiation of species. However, with the difficulty in distinguishing young ring-stage parasites, frequent misdiagnosis has been reported in areas co-endemic for *P*. *falciparum* and *P*. *vivax*[20,21]. It is well established that microscopy cannot reliably distinguish *P*. *knowlesi* from *Plasmodium malariae*[22,23], but misdiagnosis of *P*. *knowlesi* with other species may also be frequent [13]. This study therefore evaluated the accuracy of both routine hospital microscopy and microscopy performed by an experienced research microscopist, for the diagnosis of *P*. *falciparum*, *P*. *knowlesi*, and *P*. *vivax*, in an area where all three species commonly occur.

## Methods

### Study site and referral system

The study was conducted at Queen Elizabeth Hospital (QEH), an adult tertiary referral hospital in Kota Kinabalu, Sabah, Malaysia. The hospital services the West Coast and Kudat Divisions of Sabah, with six district hospitals and a population of 1.14 million. From September 2010, in response to ongoing malaria deaths in Sabah [16], new treatment and referral guidelines were instituted and included tertiary hospital referral for all malaria patients with a thick blood film reported as “4+” (indicating >10 parasites/high-power microscopy field [24]) or who had any evidence of severe malaria. Treatment was commenced prior to transfer and a pre-treatment blood film was sent with the patient. Local health clinics within the Kota Kinabalu area were required to refer all malaria patients to QEH for admission, with treatment commenced on arrival.

### Subjects

All patients referred to or admitted directly to QEH with a microscopic diagnosis of malaria were assessed for eligibility from September 2010 to October 2011 as part of a prospective study of the epidemiology, clinical spectrum and pathophysiology of knowlesi malaria, reported elsewhere [15]. Non-pregnant patients ≥12 years old were enrolled if they were within 18 hours of commencing malaria treatment, had no major co-morbidities, and had not already been enrolled in the study. Patients who were PCR negative were retrospectively excluded. Patients were classified as having severe malaria using modified 2010 WHO Severe Malaria Criteria [15,25,26]. Written informed consent was provided by patients or their relatives. Ethics approval was obtained from the Medical Research Sub-Committee of the Malaysian Ministry of Health and the Health Research Ethics Committee of the Menzies School of Health Research, Australia.

### Study procedures

Standardized data forms were used to record demographic and clinical information. Venous blood was collected in a CTAD tube labelled with the patient’s study number, and thick and thin blood smears prepared using Giemsa staining. Species identification using thick and thin blood films was performed initially by microscopists at referring district hospitals, or at QEH if presenting directly to this hospital (routine microscopy). Thick and thin films were later cross-checked by a research microscopist (cross-check microscopy) with more than 15 years’ experience, who was blinded to the results of routine microscopy. Because reliable differentiation of *P*. *knowlesi* from *P*. *malariae* is not possible [27], slides reported as *P*. *malariae*, *P*. *knowlesi*, or *P*. *malariae*/*P*. *knowlesi* were all considered a single group, further referred to as “*P*. *malariae*/*P*. *knowlesi*”. Parasite density was quantified by the research microscopist using pre-treatment slides, and reported as the number of parasites per 200 white blood cells or per 1,000 red blood cells and converted to parasites/μL using the patient’s white blood cell count or haematocrit, respectively. If a pre-treatment slide could not be reliably obtained (6% of total slides) the referring hospital’s microscopy report was used and the “1+ − 4+” grade converted into parasites/μL using the relevant median parasite density. Parasite DNA was extracted and PCR performed using previously described methods for *P*. *falciparum*, *P*. *vivax*, *Plasmodium ovale*, and *P*. *malariae*[28] and *P*. *knowlesi*[29]. PCR diagnosis was used as the gold standard. Patients were followed-up on days 14 and 28 if possible, and/or if readmitted to QEH.

### Statistical analysis

Data were analysed using STATA version 10.1 (StataCorp LP, College Station, TX, USA). For continuous variables intergroup differences were compared using the Kruskal-Wallis test, or the Mann–Whitney test for pairwise comparisons, while the χ^2^ test was used for intergroup differences between categorical variables. Logistic regression was used to assess the association between parasite count and correct identification of species.

## Results

A total of 304 patients with PCR-confirmed *Plasmodium* infection were enrolled, including 130 with *P*. *knowlesi*, 122 with *P*. *falciparum*, 43 with *P*. *vivax*, one with *P*. *malariae*, and eight with mixed-species infection. Demographic and clinical features are reported separately [15]. Half (51%) of all patients were referred from district hospitals, including 86 (66%) with knowlesi malaria, 47 (39%) with *P*. *falciparum*, and 21 (49%) with *P*. *vivax* malaria. Severe malaria occurred in 38 (29%) patients with *P*. *knowlesi*, 13 (11%) with *P*. *falciparum*, and seven (16%) with *P*. *vivax*[15]. Patients with non-severe *P*. *knowlesi* had lower parasite counts than those with non-severe *P*. *falciparum* (4,837 [IQR 1576–14,641] *vs* 10,500 [IQR 4,014–32,267] parasites/μL, p < 0.01), but not *P*. *vivax* (median 4,753 [IQR 2369–10,316] parasites/μL, p = 0.95). Among patients with severe malaria, there was no significant difference in median parasitaemia of those with *P*. *knowlesi* (80,359 [IQR 25,857–168,279] parasites/μL) and *P*. *falciparum* (72,270 [IQR 27,905–273,909] parasites/μL, p = 0.78).

Microscopy and PCR results are shown in Tables 1 and 2. Slides were unavailable for cross-check microscopy for eight (2.6%) patients. Routine and cross-check microscopy correctly identified the species in 229/304 (75%) and 242/296 (82%) patients respectively (p = 0.055), with 188/296 (64%) patients correctly diagnosed by both microscopic methods. Among patients with PCR-confirmed *P*. *knowlesi* mono-infection, routine and cross-check microscopy each correctly identified 94 (72%) patients as “*P*. *malariae*/*P*. *knowlesi*”, with 66 (51%) patients correctly diagnosed by both microscopy readings. Routine microscopy diagnosed 17 (13%) and 13 (10%) patients with PCR-confirmed *P*. *knowlesi* mono-infection as *P*. *falciparum* and *P*. *vivax* respectively, while cross-check microscopy diagnosed 28 (22%) and two (1.5%) as *P*. *falciparum* and *P*. *vivax* respectively. Four (3%) patients with PCR-confirmed *P*. *knowlesi* were diagnosed by both routine and cross-check microscopy as *P*. *falciparum*, and no patient was diagnosed by both readings as *P*. *vivax*. Among the 38 patients with severe knowlesi malaria, routine microscopy correctly diagnosed 33 (87%) as “*P*. *malariae*/*P*. *knowlesi*”. Three were diagnosed as *P*. *falciparum*, one as *P*. *vivax* and one as *P*. *falciparum*/“*P*. *malariae*/*P*. *knowlesi*”. The median parasite count among the five patients with severe knowlesi malaria and misdiagnosed by routine microscopy was 168,279 (range 26,368 – 506,218) parasites/μL, and was not significantly different to those correctly diagnosed.

**Table 1:** PCR results compared with routine microscopy

**Microscopy**	**PCR**							
	**Pf**	**Pv**	**Pk**	**Pm**	**Pf**/**Pv**	**Pf**/**Pk**	**Pf**/**Pm**	**Total**
**Pf**	110	3	17	0	2	2	0	134
**Pv**	1	23	13	0	1	0	0	38
"**Pm**/**Pk**"	8	13	94	1	0	0	1	117
"**Pm**/**Pk**"/**Pv**	0	3	2	0	0	1	0	6
"**Pm**/**Pk**"/**Pf**	2	0	3	0	0	0	0	5
**Pf**/**Pv**	1	1	0	0	1	0	0	2
“**P species**”	0	0	1	0	0	0	0	1
Total	122	43	130	1	4	3	1	304

Abbreviations: Pf = *Plasmodium falciparum*, Pv = *Plasmodium vivax*, Pk = *Plasmodium knowlesi*, Pm = *Plasmodium malariae*, P = *Plasmodium.*

**Table 2:** PCR results compared with cross-check microscopy

**Microscopy**	**PCR**							
	**Pf**	**Pv**	**Pk**	**Pm**	**Pf**/**Pv**	**Pf**/**Pk**	**Pf**/**Pm**	**Total**
**Pf**	112	0	28	0	1	3	0	144
**Pv**	1	34	2	0	1	0	0	38
"**Pm**/**Pk**"	5	3	94	1	0	0	1	104
**Pf**/**Pv**	0	1	2	0	1	0	0	4
**Po**	0	2	0	0	0	0	0	2
**Negative**	0	0	4*	0	0	0	0	4
Total	118	40	130	1	3	3	1	296

Abbreviations: Pf = *Plasmodium falciparum*, Pv = *Plasmodium vivax*, Pk = *Plasmodium knowlesi*, Pm = *Plasmodium malariae*, Po = *Plasmodium ovale*.

Note: Slides were unavailable for cross-check microscopy for eight patients. Cross-check microscopy was performed on pre-treatment slides for 280 (95%) of 296 patients.

*Includes one post-treatment slide. Parasite counts (according to routine microscopy results) of remaining three patients were 32, 55 and 1574 parasites/μL.

Among patients with PCR-confirmed *P*. *falciparum*, routine and cross-check microscopy correctly identified 110/122 (90%) and 112/118 (95%) as *P*. *falciparum*, while 8/122 (6.6%) and 5/118 (4.2%) patients respectively were diagnosed as “*P*. *malariae*/*P*. *knowlesi*”. All 13 patients with severe falciparum malaria were correctly diagnosed by routine microscopy.

Among those with PCR-confirmed *P*. *vivax*, 23/43 (53%) and 34/40 (85%) were correctly diagnosed by routine and cross-check microscopy respectively, while 13/43 (30%) and 3/40 (7.5%) patients were diagnosed as “*P*. *malariae*/*P*. *knowlesi*”. Among the 13 patients with PCR-confirmed *P*. *vivax* and misdiagnosed by routine microscopy as “*P*. *malariae*/*P*. *knowlesi*”, four (31%) were re-admitted to QEH within four months with PCR-confirmed *P*. *vivax* infection. Among the seven patients with severe vivax malaria, routine microscopy diagnosed three as *P*. *vivax*, two as “*P*. *malariae*/*P*. *knowlesi*”, one as *P*. *falciparum*/*P*. *vivax*, and one as “*P*. *malariae*/*P*. *knowlesi*”/*P*. *vivax*.

Among the eight patients with mixed-species infection by PCR, one patient with *P*. *falciparum*/*P*. *vivax* was correctly identified by routine microscopy, and another with *P*. *falciparum*/*P*. *vivax* was correctly identified by cross-check microscopy. All others were misdiagnosed by both microscopic readings (with one patient’s slide unavailable for cross-check microscopy).

An association was found between parasite count and correct identification of *P*. *vivax* by routine microscopy (OR [log increase] 1.64 [95% CI 1.01 – 2.68], p = 0.046), with a similar trend also seen with identification of *P*. *vivax* by cross-check microscopy (OR [log increase] 1.73 [95% CI 0.96 – 3.10], p = 0.067). No association however occurred between parasite count and correct identification of *P*. *knowlesi* or *P*. *falciparum*, by either routine or cross-check microscopy.

## Discussion

This study highlights the difficulties of microscopic diagnosis of *Plasmodium* species in an area where *P*. *falciparum*, *P*. *vivax* and *P*. *knowlesi* all commonly occur. Misdiagnosis of *P*. *knowlesi* as both *P*. *vivax* and *P*. *falciparum*, and *vice versa*, is common. Only 72% of patients with PCR-confirmed *P*. *knowlesi* received an accurate diagnosis by routine or by cross-check microscopy, and correlation between the microscopic methods was poor, with even fewer patients receiving an accurate diagnosis of *P*. *knowlesi* by both methods. These findings occurred despite considering *P*. *knowlesi* and *P*. *malariae* as a single group, and so were not a consequence of the well described near impossibility of distinguishing these two species. Rather, patients with PCR-confirmed *P*. *knowlesi* were commonly misdiagnosed as having either *P*. *falciparum* or *P*. *vivax* malaria, with misdiagnosis of *P*. *vivax* as “*P*. *malariae*/*P*. *knowlesi*” also common.

The difficulty with distinguishing *P*. *knowlesi* from *P*. *falciparum* by microscopy has been previously described, due to similarities between the young rings of *P*. *knowlesi* and ring forms of *P*. *falciparum*, including double chromatin dots, multiple-infected erythrocytes and applique forms [22,23]. In a previous study in Sarawak, 11/216 (5%) patients diagnosed by microscopy as “*P*. *malariae*” were actually *P*. *falciparum* by PCR, and 33/312 (11%) microscopy-diagnosed *P*. *falciparum* cases were *P*. *knowlesi* by PCR [13]. In this previous study and in the current study, however, the difficulty differentiating *P*. *knowlesi* and *P*. *vivax* was also notable. In the current study 30% of patients with PCR-confirmed *P*. *vivax* were misdiagnosed by routine microscopy as “*P*. *malariae*/*P*. *knowlesi*”, with four patients subsequently re-admitted with presumed vivax relapses due to lack of primaquine treatment. In the Sarawak study, 43 of 440 (10%) patients with PCR-confirmed *P*. *vivax* malaria were misdiagnosed as “*P*. *malariae*” [13]. In a series of malaria deaths in Sabah, one of six fatal cases of *P*. *knowlesi* was misdiagnosed as *P*. *vivax* by microscopy and a fatal case of *P*. *vivax* was misdiagnosed as “*P*. *malariae*” [18].

This frequent misdiagnosis of *P*. *vivax* as “*P*. *malariae*/*P*. *knowlesi*” has significant implications for malaria control, as failure to administer anti-hypnozoite treatment may lead to increased transmission and may hamper efforts to eliminate vivax malaria in regions where *P*. *knowlesi* is common. These findings support the current Sabah Ministry of Health policy for the performance of reference centre PCR on all patients with a microscopic diagnosis of “*P*. *malariae*/*P*. *knowlesi*” to enable administration of primaquine to those found to have misdiagnosed *P*. *vivax*. In knowlesi-endemic areas, where logistically possible, PCR should also be performed on at least a proportion of slides diagnosed as *P*. *falciparum* or *P*. *vivax*, to allow monitoring of the accuracy of microscopic diagnoses at different clinical sites, and to identify areas where additional training of microscopists may be required. Given the inaccuracies of microscopic diagnosis, performance of PCR is also essential to maintain accurate surveillance, particularly monitoring the emergence of *P*. *knowlesi*.

The inaccuracy of microscopy for differentiating *Plasmodium* species creates difficulties with basing treatment decisions on microscopic results. The 2008 Malaysian Ministry of Health malaria treatment guidelines recommend chloroquine for uncomplicated *P*. *malariae*/*P*. *knowlesi* malaria; chloroquine plus primaquine for uncomplicated *P*. *vivax* malaria; artemisinin combination treatment (ACT; artesunate/mefloquine [Artequine®] or artemether/lumefantrine [Riamet®]) for uncomplicated *P*. *falciparum* malaria; and intravenous artesunate, or intravenous quinine plus oral doxycycline, for severe *P*. *falciparum* or severe *P*. *malariae*/*P*. *knowlesi* malaria [30]. No recommendations are given for treatment of severe vivax malaria. At Queen Elizabeth Hospital and referral hospitals in its catchment area, updated treatment guidelines recommend ACT for uncomplicated *P*. *falciparum* or *P*. *malariae*/*P*. *knowlesi* malaria; chloroquine plus primaquine, or ACT, for uncomplicated *P*. *vivax* malaria; and intravenous artesunate (followed by oral therapy as above) for severe malaria from any species [15].

Given the different treatment recommendations for each species in both of these guidelines, inappropriate treatment decisions may be made as a result of incorrect microscopic diagnoses. Misdiagnosis of *P*. *falciparum* as *P*. *knowlesi* would, under current national guidelines, result in administration of chloroquine for *P*. *falciparum*. Given widespread resistance of *P*. *falciparum* to chloroquine, this would lead to increased risk of complications and/or fatal outcome, as well as increased transmission. Patients with severe *P*. *knowlesi* malaria misdiagnosed as *P*. *vivax* may fail to receive immediate parenteral treatment if national guidelines are followed, and this scenario has been previously associated with fatal outcomes [18]. Even if the updated hospital guidelines are followed, misdiagnosis of severe *P*. *knowlesi* as *P*. *vivax* may still lead to treatment with oral chloroquine if signs of severity are missed, potentially leading to adverse outcomes given the slower parasite clearance associated with chloroquine as compared to oral ACT [16].

These results therefore support the argument for a unified treatment strategy of ACT for uncomplicated malaria from all *Plasmodium* species in knowlesi-endemic areas, an approach increasingly recommended for regions co-endemic for *P*. *falciparum* and *P*. *vivax*[31]. These data also support the 2012 WHO recommendation for intravenous artesunate to be given to any patient meeting severe malaria criteria [25]. Even if signs of severity are overlooked among patients with knowlesi malaria, treatment with oral ACT may ensure more rapid parasite clearance and may lead to improved outcomes compared to treatment with oral chloroquine [16], although the optimal oral agent for uncomplicated *P*.*knowlesi* remains undetermined.

An additional advantage of this unified treatment approach would be the avoidance of inadvertent use of chloroquine for *P*. *falciparum* misdiagnosed as another species. Furthermore, chloroquine-resistant *P*. *vivax* is an increasing problem throughout Southeast Asia [31], and has been previously documented in Sabah [32] and Peninsular Malaysia [33-35]. Use of chloroquine for *P*. *vivax* malaria may therefore be associated with treatment failures, and may potentiate spread of chloroquine resistance.

Finally, this study highlights the need to develop rapid diagnostic tests (RDTs) that have the ability to distinguish between *Plasmodium* species, in order that reliable results can be obtained more quickly and cheaply than is possible with PCR. Although histidine-rich protein 2 (HRP2)-based RDTs are able to diagnose *P*. *falciparum*, RDTs that distinguish *P*. *knowlesi* from *P*. *vivax* are not yet available. Limited data suggest that *P*. *knowlesi* cross-reacts with both *P*. *falciparum* and *P*. *vivax*-specific pLDH [36-39], and RDTs that combine these antigens with HRP2 may therefore allow differentiation between *P*. *vivax*, *P*. *falciparum* and *P*. *knowlesi* mono-infections. However, while a pLDH RDT has shown high sensitivity for the diagnosis of severe malaria from all three of these species, neither pLDH- or aldolase-based RDTs have demonstrated sufficiently high sensitivity for uncomplicated *P. knowlesi*[40]. Prospective evaluation of more sensitive RDTs in knowlesi-endemic areas is needed.

This study has found that microscopy does not reliably distinguish between *P*. *falciparum*, *P*. *vivax* and *P*. *knowlesi* in areas co-endemic for all three species, with misdiagnosis of *P*. *vivax* and *P*. *knowlesi* particularly common. In *P*. *knowlesi*-endemic areas these limitations of microscopic diagnosis must be considered when developing strategies to monitor the prevalence of the different malaria species, and when developing treatment guidelines.

## Competing interests

The authors declare that they have no competing interests.

## Authors’ contributions

NMA, TWY, TW and BEB conceived and designed the study; BEB collected and analysed the data. BEB and NMA wrote the manuscript. MJG assisted with data collection. All authors read and approved the final manuscript.
